# AMP–Chitosan Coating with Bactericidal Activity in the Presence of Human Plasma Proteins

**DOI:** 10.3390/molecules25133046

**Published:** 2020-07-03

**Authors:** Cláudia Monteiro, Hélia Fernandes, Diana Oliveira, Nuno Vale, Mariana Barbosa, Paula Gomes, M. Cristina L. Martins

**Affiliations:** 1 i3S, Instituto de Investigação e Inovação em Saúde, Universidade do Porto, Rua Alfredo Allen, 208, 4200-135 Porto, Portugal; claudia.monteiro@ineb.up.pt (C.M.); heliajp@hotmail.com (H.F.); dianarosalopesoliveira@gmail.com (D.O.); marianamoreirabarbosa@gmail.com (M.B.); 2 INEB, Instituto de Engenharia Biomédica, Universidade do Porto, Rua Alfredo Allen, 208, 4200-135 Porto, Portugal; 3 UCIBIO-REQUIMTE, Departamento de Química e Bioquímica, Faculdade de Ciências, Universidade do Porto, Rua do Campo Alegre, 4169-007 Porto, Portugal; nuno.vale@ff.up.pt; 4 LAQV-REQUIMTE, Departamento de Química e Bioquímica, Faculdade de Ciências, University of Porto, Rua do Campo Alegre, 4169-007 Porto, Portugal; pgomes@fc.up.pt; 5 Instituto de Ciências Biomédicas Abel Salazar, Universidade do Porto, Rua de Jorge Viterbo Ferreira, 228, 4050-313 Porto, Portugal

**Keywords:** antimicrobial peptides, biomaterials, chitosan, coatings, human plasma

## Abstract

Antibiotic resistance is increasing and new strategies are needed to fight infection. Advanced materials are promising tools that can be combined with innovative alternatives to conventional antibiotics to allow more targeted and efficient treatment. In this work, we explored the activity against *Staphylococcus epidermidis* (*S. epidermidis*) of the α-helical antimicrobial peptide (AMP) MSI-78(4-20) (KFLKKAKKFGKAFVKIL) when covalently bound to a chitosan coating. The AMP MSI-78(4-20) (17 mer) is an improved version of its parent MSI-78 (22 mer; commercially known as Pexiganan), a cost-effective short AMP, which was demonstrated to be as effective as MSI-78 and less toxic to eukaryotic cells. An MSI-78(4-20)–chitosan coating could be applied in several infection scenarios, ranging from bone implants to wound dressings, as chitosan possesses osteoconductive and hemostatic properties. Cysteine-modified MSI-78(4-20) was covalently immobilized onto the chitosan coating through a succinimidyl-[(*N*-maleimidopropionamido)-octaethyleneglycol] ester (SM(PEG)_8_), a heterobifuncional crosslinker, with *N*-hydroxysuccinimide (NHS) ester and maleimide groups, by its *N*- and *C*- termini. The MSI-78(4-20)–chitosan coating demonstrated bactericidal properties independently of the tethering site and an improved performance in the presence of plasma proteins, which mimics conditions that will be encountered in vivo. This AMP–chitosan coating has therefore great potential for applications in medical devices such as implants or even wound dressings.

## 1. Introduction

Microbial infections caused by antibiotic-resistant strains are responsible for large numbers of deaths worldwide, and it has been estimated that ten million people will die annually by 2050 in the USA alone if alternatives are not found [1]. Apart from specific resistance mechanisms, bacteria can grow in highly organized communities that form biofilms, which are extremely resistant to conventional antibiotics. This mode of growth may occur on a surface such as an implant or a catheter, or at any place in the body where host defenses are compromised (e.g., chronic wounds with impaired blood supply) [2]. Advanced materials such as coatings, hydrogels, and nanoparticles have been combined with antimicrobial compounds to tackle these problems [3]. Among other strategies, antimicrobial peptides (AMPs) have shown promising results in the development of broad-spectrum antibacterial materials and surfaces [3].

AMPs are oligopeptides, active against a broad range of bacteria, fungi, viruses, and parasites, and usually possessing an overall positive charge and an amphipathic structure. Most AMPs, namely cationic AMPs, act via nonspecific interactions with negatively charged bacterial membranes, causing membrane disruption or trans-membrane pore formation, ultimately leading to cell lysis and death [4]. This mode of action makes resistance to AMPs very unlikely, as bacteria would have to remodel their membrane, which is too costly for most strains [5]. All these characteristics make AMPs an excellent choice for the development of antimicrobial materials and coatings. 

In this work, we explored the activity of the α-helical AMP MSI-78(4-20) (KFLKKAKKFGKAFVKIL) when covalently bound to a chitosan coating against *Staphylococcus epidermidis* (*S. epidermidis*). The AMP MSI-78(4-20) (17 mer) is an improved version of its parent MSI-78 (GIGKFLKKAKKFGKAFVKILKK) (22 mer), i.e., it is a cost-effective short AMP that has been demonstrated to be equally potent and less toxic to eukaryotic cells than its parent peptide [6]. Due to their versatility, chitosan coatings may be applied in several infection scenarios, ranging from bone implants, due to their osteoconductive characteristics, to wound dressings, as chitosan possesses excellent hemostatic properties [7]. *S. epidermidis* was chosen as it is a strain highly prevalent in the human skin that becomes an opportunistic pathogen in wound- and implant-associated infections.

Covalent immobilization of an AMP is a very interesting approach for the application of AMPs in coatings as it provides good surface availability and homogeneous distribution of the peptide on the surface while at the same time reducing enzymatic degradation, thus increasing long-term stability and avoiding the toxicity associated with the application of high AMP concentrations [4,8,9]. However, AMPs’ activity may be influenced by several factors, such as solid supports, spacer specificities, surface density, exposure, orientation, and peptide stability [4]. Several α-helical AMPs have already been immobilized on different surfaces using a variety of conditions, and results show that their efficacy may depend on the terminus chosen to tether the peptide to the surface, as this may lead to exposure of termini with different characteristics or induce different AMP conformations and orientation on the surface [10,11,12,13]. MSI-78, the parent peptide, was previously immobilized on alkyne-terminated self-assembled monolayers through both its *C*- and *N*- termini. Results showed that immobilization through the *N*-terminus produced slightly better antimicrobial activity, killing bacterial cells more quickly [13].

The AMP, surface characteristics, the chemical strategy for AMP immobilization, and the biological environment define the antimicrobial performance of these developed coatings; therefore, it is essential to study AMPs’ activity upon immobilization in different orientations under the specific conditions in which the AMPs will be applied.

## 2. Results

### 2.1. Surface Characterization

**Figure 1:**
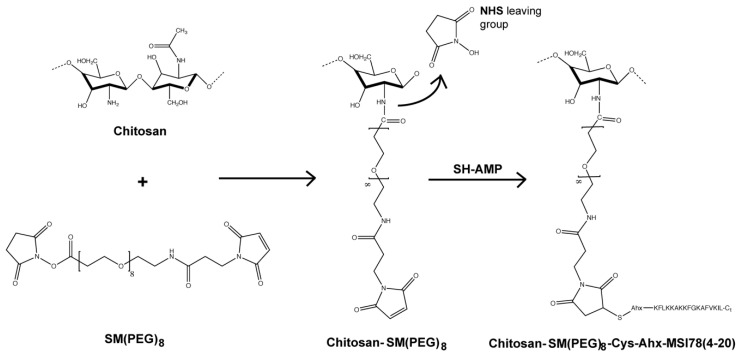
Route taken to graft MSI-78(4–20) onto chitosan. Conjugation was performed using the succinimidyl-[(*N*-maleimidopropionamido)-octaethyleneglycol] ester (SM(PEG)_8_) heterobifuncional crosslinker bearing an N-hydroxysuccinimide moiety that allows covalent conjugation with the amines of chitosan, and a maleimide moiety for chemoselective conjugation with the sulfhydryl group of the cysteine residue in the peptide.

**Figure 2:**
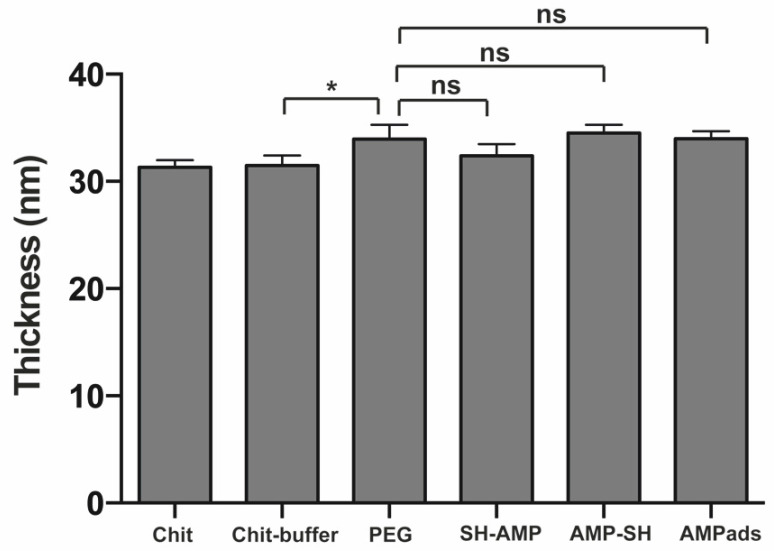
Thickness of the unmodified chitosan films, chitosan (Chit) and chitosan exposed to reaction buffer (Chit-buffer); chitosan films modified with succinimidyl-[(*N*-maleimidopropionamido)-octaethyleneglycol] ester (SM(PEG)_8_) (PEG); chitosan films modified with AMP (SH-AMP/AMP-SH), and adsorption control (AMPads), according to ellipsometry measurements. Statistically significant differences at *p* < 0.05 are indicated with *; ns, not significant.

**Figure 3:**
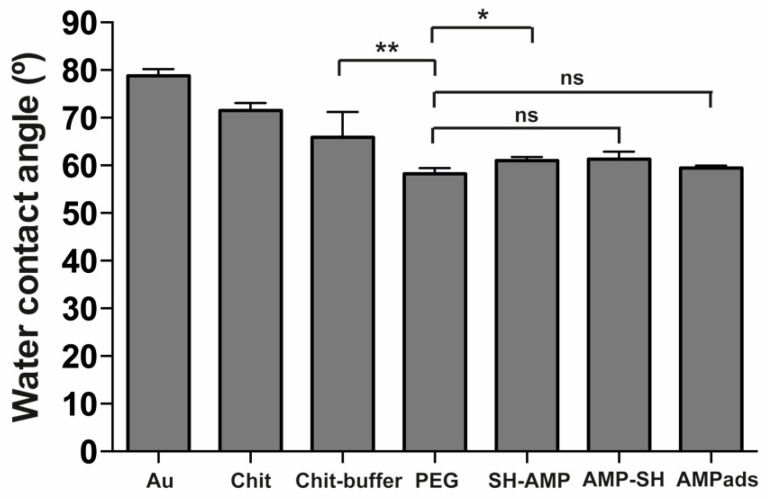
Wettability of the unmodified chitosan films, chitosan (Chit) and chitosan exposed to reaction buffer (Chit-buffer); chitosan films modified with succinimidyl-[(*N*-maleimidopropionamido)-octaethyleneglycol] ester (SM(PEG)_8_) (PEG); chitosan films modified with AMP (SH-AMP/AMP-SH), and adsorption control (AMPads), according to contact angle measurements. Statistically significant differences are indicated as * *p* < 0.05, ** *p* < 0.01; ns, not significant.

MSI-78(4-20) was covalently immobilized on the chitosan film using cysteine-modified MSI-78(4-20) derivatives, where the cysteine was added to either the *N-* (SH-AMP) or the *C-* (AMP-SH) termini of the native sequence. Immobilization onto chitosan was achieved through the heterobifunctional crosslinker succinimidyl-[(*N*-maleimidopropionamido)-octaethyleneglycol] ester (SM(PEG)_8_), which is based on a poly(ethylene glycol)_8_ (PEG)_8_ core functionalized with *N*-hydroxysuccinimide (NHS) at one end, and with a maleimide at the other (Figure 1). Surfaces were characterized in terms of thickness (ellipsometry) and wettability (water contact angle). Ellipsometry measurements (Figure 2) showed a significant increase of about 2.5 nm in surface thickness after PEG immobilization. Knowing that the spacer arm was 3.9 nm in length, these results suggested a successful functionalization. After AMP immobilization, no significant differences in thickness were observed. Regarding wettability (Figure 3), the chitosan film presented a water contact angle of 66 ± 5°, in agreement with previously published results [14,15]. After PEG immobilization, the contact angle decreased significantly (58 ± 1°), as expected due to the well-known hydrophilic character of PEG [16]. After peptide immobilization, there was only a slight increase in the water contact angle for both SH-AMP (61 ± 1°) and AMP-SH (61 ± 2°).

### 2.2. Antimicrobial Activity

**Figure 4:**
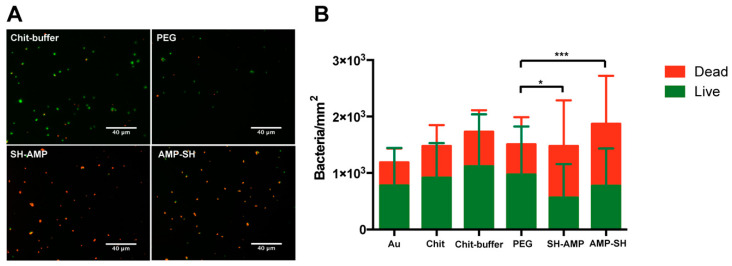
Antimicrobial activity of MSI-78(4-20)–chitosan films. (**A**) Representative images of chitosan films labeled with LIVE/DEAD^™^ BacLight ^™^ Bacterial Viability Kit (Thermo Fisher Scientific, Waltham, Massachusetts), after contact with *S. epidermidis*. Images were collected using an inverted fluorescence microscope with 400× magnification. (**B**) Quantification of the viability of adherent bacteria. Statistically significant differences are indicated as * *p* < 0.05, *** *p* < 0.001 (non-parametric Kruskal–Wallis test of dead group). Gold substrate (Au); unmodified chitosan films, chitosan (Chit) and chitosan exposed to reaction buffer (Chit-buffer); chitosan films modified with succinimidyl-[(*N*-maleimidopropionamido)-octaethyleneglycol] ester (SM(PEG)_8_) (PEG); chitosan films modified with AMP (SH-AMP /AMP-SH).

**Figure 5:**
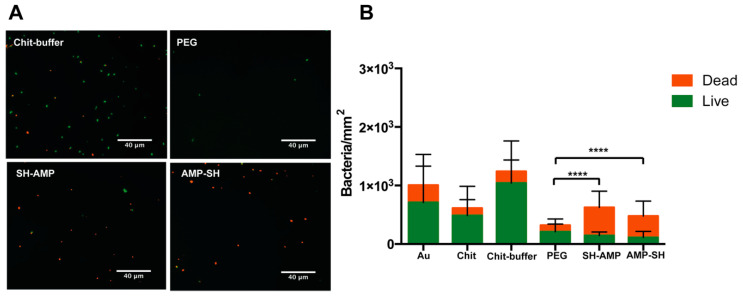
Antimicrobial activity of MSI-78(4-20) chitosan films in 1% plasma. (**A**) Representative images of chitosan films labeled with LIVE/DEAD^™^ BacLight ^™^ Bacterial Viability Kit (Thermo Fisher Scientific, Waltham, Massachusetts), after contact with *S. epidermidis*. Images were collected using an inverted fluorescence microscope with 400x magnification. (**B**) Quantification of the viability of adherent bacteria. Statistically significant differences are indicated with **** (*p* < 0.0001) (non-parametric Kruskal–Wallis test of dead group). Gold substrate (Au); unmodified chitosan films, chitosan (Chit) and chitosan exposed to reaction buffer (Chit-buffer); chitosan films modified with succinimidyl-[(*N*-maleimidopropionamido)-octaethyleneglycol] ester (SM(PEG)_8_) (PEG); chitosan films modified with AMP (SH-AMP /AMP-SH).

The antimicrobial activity of MSI-78(4-20)–chitosan film was assessed by determination of bacterial adhesion and viability of *S. epidermidis* in phosphate-buffered saline (PBS) and PBS supplemented with 1% human plasma (Figure 4 and Figure 5). Regarding the results in PBS (Figure 4), bacterial adhesion did not show significant alterations between samples. Concerning viability, chitosan films killed around 35% of bacteria (Chit 38%; Chit-buffer 35%). For surfaces with immobilized peptide, whether in the SH-AMP or the AMP-SH orientation, similar and high percentages of bacterial death were observed (SH-AMP 62%; AMP-SH 58%). 

In the presence of human plasma (Figure 5), a significant decrease in bacterial adhesion was observed in the PEG-modified surface, a reduction of approximately 75% when comparing to Chit-buffer films (*p* < 0.0001, non-parametric Kruskal–Wallis test). Upon AMP immobilization, a slight increase in bacterial adhesion was observed; however, adhesion remained significantly reduced when compared to Chit-buffer films, showing a reduction of approximately 50% (*p* = 0.0027) and 65% (*p* < 0.001) in the SH-AMP and AMP-SH orientations, respectively (non-parametric Kruskal–Wallis test). Regarding viability, chitosan films killed only approximately 20% of bacteria (Chit, 21%; Chit-buffer, 16%), while MSI-78(4-20)–chitosan films killed 76% of adherent bacteria independently of the grafting orientation.

## 3. Discussion

In this work, we tested the activity of MSI-78(4-20) when covalently immobilized onto a chitosan coating through a PEG spacer by either its *N*- or its *C*- terminus. Moreover, we studied the AMP coating’s effect in the presence of human plasma proteins, mimicking conditions to be encountered in vivo. To this end, we began by producing chitosan films by spin-coating, and then grafted the peptide onto the film according to the route depicted in Figure 1. A PEG linker with 8 ethylene glycol units was chosen based on previous studies where PEG linkers that were too short or too long were found to be deleterious for the structural and functional features of tethered peptides, with 6-18 ethylene glycol units apparently allowing for a better AMP performance [11,17].

Surfaces were characterized in terms of thickness (ellipsometry) and wettability (water contact angle). Ellipsometry measurements (Figure 2) showed successful PEG functionalization. The slight discrepancy between the observed surface thickness and the PEG length may have been due to PEG flexibility or to the fact that the value obtained was the average thickness of a given surface area, which may not have been completely covered by PEG. After AMP immobilization, no significant differences in thickness were observed. These results can be explained by the spacer’s and peptide’s flexibility, which can result in different orientations towards the chitosan films. Regarding wettability (Figure 3), a successful PEG functionalization was demonstrated. After peptide immobilization, only a slight increase in the water contact angle was observed, which was likely due to the presence of amino acids of hydrophobic and hydrophilic character distributed along the peptide sequence, which led to minor differences in wettability.

MSI-78(4-20)–chitosan films demonstrated efficient antimicrobial effects against *S. epidermidis* in both PBS and PBS supplemented with 1% human plasma (Figure 4 and Figure 5). Regarding the results in PBS, for the PEG control sample, a possible decrease in adhesion was not observed, but the presence of maleimide exposed in the linker might have altered the non-fouling characteristics of PEG. Concerning viability, chitosan films killed around 35% of bacteria (Chit 38%; Chit-buffer 35%). Despite the inherent antimicrobial activity of chitosan, its efficiency is often suboptimal, depending on the degree of acetylation and molecular weight. As such, several authors have studied different strategies to improve the antimicrobial efficiency of chitosan films [18,19,20]. Surfaces onto which either SH-AMP or AMP-SH derivatives of MSI-78(4-20) were grafted induced similar and high percentages of bacterial death (SH-AMP 62%; AMP-SH 58%), demonstrating that this peptide retained activity after immobilization on chitosan films through either the *N*- or the *C*-terminus. Thus, attachment site of the α-helical MSI-78(4-20) did not seem to influence AMP antimicrobial efficiency. The *C*-terminus of MSI-78(4-20) is slightly more hydrophobic than the *N*-terminus; however, this was not detectable after their surface immobilization, since no differences in surface wettability were observed between surfaces where MSI-78(4-20) was immobilized in different orientations (Figure 3). In line with these observations, Han et al. showed that despite AMP CP1 acquiring different conformations and orientations upon immobilization through the *N*- and *C*-termini, a possible alteration of conformation and orientation might be induced upon contact with bacteria, leading to similar antimicrobial activity [11].

Other authors have evaluated the antimicrobial activity exhibited by different surfaces after AMP tethering; however, such studies seldom have addressed conditions that mimic those in the human body. Thus, we evaluated bacterial adhesion and viability upon contact with the AMP-grafted surfaces in the presence of human plasma (Figure 5). Regarding bacterial adhesion, a significant decrease was observed in the PEG-modified surface. It is possible that non-adhesive plasma proteins such as albumin adsorb to the PEG terminal maleimides, making the non-fouling effect of PEG more evident. Upon AMP immobilization, although there was an increase of bacterial adhesion when compared to the PEG-modified film, probably due to electrostatic attraction between the cationic AMP and the bacteria, MSI-78(4-20)–chitosan films maintained a significantly low bacterial adhesion, while killing a high percentage of adherent bacteria (76%) independently of the grafting orientation. In contrast, chitosan films seemed to lose antimicrobial capacity in the presence of plasma, killing only approximately 20% of bacteria (Chit, 21%; Chit-buffer, 16%), which could be related to the adsorption of plasma proteins onto the chitosan film surface, as previously demonstrated [21]. 

AMP-grafted chitosan coatings were previously explored by our group, using hLF(1-11) and Dhvar-5 as reference wide-spectrum AMPs. Costa et al. and Barbosa et al. showed the importance of peptide attachment site in covalent immobilization on antimicrobial activity [14,15,22]. hLF(1-11) and Dhvar-5 possess a head-to-tail amphipathicity that probably promotes distinctive interactions with bacteria. In this work, we showed that the antimicrobial activity of the α-helical MSI-78(4-20) peptide was not affected by attachment site upon immobilization onto chitosan films, probably due to the distribution of the hydrophobic and hydrophilic amino acids along the sequence. Moreover, the fact that, after surface grafting, the antimicrobial efficacy was not affected by plasma proteins is also a remarkable observation, since inactivation of AMPs by anionic plasma proteins is a major concern. Indeed, Huang et al. demonstrated that in solution, cationic α-helical AMPs can bind with different affinities to proteins, which may result in reduced peptide biological activity [23]. However, after surface immobilization through a PEG spacer, it is possible that plasma proteins may not efficiently adsorb to AMPs, as most proteins adsorb onto surfaces as monolayers, generating close-packed formations [24]. Most probably, proteins adsorb at the exposed chitosan surface, leaving protruding AMPs free to interact with anionic bacteria.

## 4. Materials and Methods 

### 4.1. Peptides Synthesis and Characterization

Peptides MSI-78(4-20) (KFLKKAKKFGKAFVKIL), Cys-Ahx-MSI-78(4-20) (SH-AMP), and MSI-78(4-20)-Ahx-Cys (AMP-SH) were produced via Fmoc/tBu solid-phase peptide synthesis methodologies assisted with microwave energy (Liberty 1 Microwave Peptide Synthesizer, CEM Corporation, Matthews, NC, USA). Crude products were purified by reverse-phase liquid chromatography and confirmed by high-performance liquid chromatography (Hitachi-Merck LaChrom Elite, Chiyoda-ku, Tokyo, Japan), liquid chromatography–electrospray ionization mass spectrometry (LCQ-DecaXP LC-MS system, ThermoFinnigan, San Jose, CA, USA), and ultraviolet spectrometry. The peptides used presented a purity level of at least 95%.

### 4.2. Surface Preparation

#### 4.2.1. Substrate Preparation

Gold (Au) substrates (1 × 1 cm) obtained from Instituto de Engenharia de Sistemas e Computadores—Microsistemas e Nanotecnologias, Portugal (INESC-MN) were prepared by deposition of thin layers of chromium (2.3 nm) and gold (37 nm) onto silicon wafers. Before use, substrates were washed to remove the photoresistant and organic residues, as described previously [25]. Briefly, substrates were submerged in acetone (Merck, Kenilworth, New Jersey, NJ, USA), sonicated, and then washed with ethanol (AGA, Lisbon, Portugal) and immersed in “piranha” solution (7 parts concentrated H_2_SO_4_ and 3 parts 30% H_2_O_2_) for 5 min (caution: this solution reacts violently with many organic materials and should be handled with great care). Substrates were cleansed sequentially with ethanol, water type 2, and ethanol, followed by drying with a gentle stream of argon.

#### 4.2.2. Preparation of Ultrathin Chitosan Films

Ultrathin chitosan films were produced as previously described [26]. Squid-pen chitosan with a molecular weight higher than 5 × 10^5^ Dalton and a deacetylation degree (DD%) of ~95% (France Chitine) was purified by the re-precipitation method and analyzed by Fourier Infrared Spectroscopy (FTIR). Ultrathin chitosan films were prepared by spin-coating (Laurell Technologies Corporation, North Wales): 150 μL of chitosan solution (0.4% in acetic acid *w*/*v*) was deposited on Au substrates twice successively and spun at 9000 rpm for 1 min. The ultrathin films prepared were then neutralized with 0.1 M sodium hydroxide (NaOH), rinsed with Milli-Q water, and dried with a gentle stream of argon until further use.

#### 4.2.3. MSI-78(4-20) Immobilization on Chitosan Films

Peptide immobilization on chitosan films (Figure 1) was done using an amide-to-sulfhydryl crosslinker with a polyethylene glycol (PEG) spacer arm, the succinimidyl-(*N*-maleimidopropionamido)-octaethyleneglycol ester, SM(PEG)_8_, with an arm of 3.925 nm and a MW of 689.71 g/mol, obtained from (Thermo Fisher Scientific, Waltham, MA, USA). First, SM(PEG)_8_ was reacted with the chitosan film; to this end, chitosan films were hydrated for 30 min under slow stirring; SM(PEG)_8_ was then incubated with the films in phosphate buffer, pH = 7.2, at 4 °C for 24 h and added gradually to the films, reaching a final concentration of 10 mM without considering hydrolysis. After incubation, substrates were washed with Milli-Q water and sonicated in an ultrasonic bath for 1 min to ensure the removal of SM(PEG)_8_ not covalently bound to the surface. 

SH-AMP or AMP-SH was treated with tris(2-carboxyethyl)phosphine (TCEP) (1.1 eq), under slow stirring for 1 h. TCEP is a reducing agent able to reduce peptide disulfide bounds, so that reduced sulfhydryls were available to react with the maleimide moiety of the crosslinker. The peptides were reacted with the PEG-modified chitosan in phosphate buffer at pH = 6.6 for 24 h, under slow stirring at 4 °C, and at a concentration of 1 mg/mL. After incubation, surfaces were washed with Milli-Q water and sonicated for 1 min in an ultrasonic bath to ensure removal of peptide not covalently bound to the surface. Finally, samples were dried with a gentle stream of argon and stored until further use. Surfaces were characterized in terms of thickness (ellipsometry) and wettability (water contact angle).

### 4.3. Surface Characterization

#### 4.3.1. Ellipsometry

Samples were dried in a vacuum oven at room temperature for at least 1 h before measurements. Ellipsometry measurements were performed using an imaging ellipsometer, model EP^3^, from Nanofilm Surface Analysis, operated in polarizer-compensator-sample-analyzer (PCSA) mode (null ellipsometry). The light source was a solid-state laser with a wavelength of 532 nm. The gold substrate refractive index (*n* = 0.70) and extinction coefficient (k = 2.63) were determined using a delta and psi spectrum with an angle variation between 63° and 73°. The thickness of the chitosan films was determined considering *n* = 1.54 and k = 0 for the chitosan film [27]. Results are presented as the average of two measurements on three independent samples.

#### 4.3.2. Contact Angle Measurement

All samples were dried at room temperature in a vacuum oven for at least 1 h before measurements. Measurements were performed using the sessile-drop method with a contact angle measuring system from (Data Physics, San Jose, CA, USA), model optical contact angle 15 (OCA 15), equipped with a video charge-coupled device (CCD) camera, and sca 20 software [25]. Images were taken every 2 s over 3 min after deposition of a 4 μL drop of Milli-Q water. Droplet profiles were fitted using the Young–Laplace mathematical function. The water contact angle of each sample was calculated by extrapolating the time-dependent curve to zero. Results are the average of two measurements on three independent samples.

### 4.4. Antimicrobial Activity

#### 4.4.1. Bacterial Strains and Growth Conditions 

*S. epidermidis* (ATCC 35984) was obtained from American Type Culture Collection. Bacteria were grown overnight at 37 °C on tryptic soy agar (TSA), (Merck, Kenilworth, NJ, USA) or on tryptic soy broth (TSB), (Merck, Kenilworth, NJ, USA) at 150 rpm. Inoculum was adjusted by measuring optical density (OD) at 600 nm and confirmed by colony-forming unit (CFU) counts.

#### 4.4.2. Adhesion and Viability Test

Surfaces were washed twice in ethanol (70%) for 30 min and thrice in sterile phosphate-buffered saline (PBS). Samples were dried in a sterile environment and then transferred to a 24 well plate (Sarstedt, Ltd., Newton, MA, USA). Antimicrobial activity was evaluated with a bacterial adhesion assay adapted from Pallavicini et al. [28]. A 5 μL drop of bacterial suspension with an approximate concentration of 1 × 10^8^ CFUs/mL in PBS or 1% plasma in PBS was dispensed onto each sample and covered with a sterile glass coverslip with a diameter of 1 cm^2^ to facilitate contact between the surface and bacteria. The samples were then incubated at 37 °C for 5 h in a wet environment. Viability of adherent bacteria was assessed using the LIVE/DEAD^™^ BacLight ^™^ Bacterial Viability Kit (Thermo Fisher Scientific, Waltham, MA, USA). After removal of non-adherent bacteria, surfaces were washed twice with PBS and once with 0.85% sodium chloride (NaCl). Surfaces were then stained with a combination of two dyes, red-fluorescent propidium iodide (PI) and green-fluorescent syto 9, for 15 min at room temperature, protected from light. PI labeled only dead bacteria, whereas the syto 9 labeled live bacteria. Finally, surfaces were mounted on slides using VECTASHIELD^®^ mounting medium for microscopy observation. Images were obtained with an inverted fluorescence microscope (Axiovert 200 M, Zeiss, Germany) using a 400× magnification. To quantify the total adherent bacteria, five fields of each sample were obtained and analyzed using ImageJ software. Results are presented as bacteria per unit area; three replicates for each condition were analyzed in two independent assays.

### 4.5. Statistical Analysis 

Statistical analysis was performed using ANOVA or non-parametric Kruskal–Wallis test. Data are expressed as the mean ± standard deviation (SD) and p values <0.05 were considered significant, with * corresponding to *p* < 0.05, **corresponding to *p* < 0.01, *** corresponding to *p* < 0.001, and **** corresponding to *p* < 0.0001.

## 5. Conclusions

The MSI-78(4-20)–chitosan coating demonstrated excellent bactericidal properties while inducing low bacterial adhesion in the presence of human plasma proteins, therefore suggesting great potential for application in a variety of medical devices such as implants or wound dressings.
